# P26 Evaluating the antibacterial activity of recombinant phage-encoded lysin against drug-resistant uropathogenic *Escherichia coli*

**DOI:** 10.1093/jacamr/dlad077.030

**Published:** 2023-08-02

**Authors:** Sakshi Dhar, Sonia Nain, Vaishali Gupta, Utkarsh Gaharwar, Bhakti Chavan, Ritam Das, Sarmad Hanif, Urmi Bajpai, Deepa Sikriwal, Aasiya Choudhary, Safia Syeda, Sanket Shah, Syed Ahmed

**Affiliations:** Techinvention Lifecare Private Limited, Mumbai, India; Techinvention Lifecare Private Limited, Mumbai, India; Techinvention Lifecare Private Limited, Mumbai, India; Techinvention Lifecare Private Limited, Mumbai, India; Techinvention Lifecare Private Limited, Mumbai, India; Techinvention Lifecare Private Limited, Mumbai, India; Techinvention Lifecare Private Limited, Mumbai, India; Acharya Narendra Dev College, University of Delhi, New Delhi, India; Techinvention Lifecare Private Limited, Mumbai, India; Techinvention Lifecare Private Limited, Mumbai, India; Techinvention Lifecare Private Limited, Mumbai, India; Techinvention Lifecare Private Limited, Mumbai, India; Techinvention Lifecare Private Limited, Mumbai, India

## Abstract

**Background:**

Atrocious use of antibiotics has led to the emergence of MDR in uropathogenic *Escherichia coli* (UPEC) that poses a serious challenge in the management of urinary tract infections (UTIs). The WHO has described antibiotic resistance in uropathogens as a key pressure point in the burgeoning global antimicrobial resistance crisis. Given this grim situation, we are exploring phage-encoded lysins as plausible alternatives.

**Objectives:**

Our study involved an *in silico* strategy for the discovery and characterization of lysin sequences (seq) targeting *E. coli* cell wall and evaluating the bactericidal activity of these recombinant lysins using *in-vitro* assays.

**Methods:**

Novel lysin sequences were searched by BLAST homology and by screening *E. coli* prophages in the database (using PHASTER). Lysozyme-like domain was observed in 9 out of 16 lysins. Their characterization depicted modular or globular structure. Based on the physicochemical properties, 7 out of 16 lysins were selected for cloning, expression, and purification as recombinant proteins for evaluating the bactericidal activity.

**Results:**

Among the several lysins screened, lysin seq 5 demonstrated the highest activity using *in vitro* assays. Using static biofilm assay, lysin seq 5 (180 μg) showed efficient reduction (>50%) in the biofilm formed by ATCC UPEC 700928 strain. As per turbidity reduction method, lysin seq 5 (50 μg) showed 37% drop in OD_600nm_ on UPEC 700928 strain after 3 h of incubation at 37°C. Using spot on lawn assay, lysin seq 5 (20 μg) exhibited lytic activity (zone of inhibition) on five drug-resistant clinical UTI isolates, which were pretreated with an outer membrane permeabilizer (OMP), viz., EDTA (0.3 mM).

**Conclusions:**

Lysin seq 5 exhibited antibiofilm activity against UPEC 700928 strain as well as lytic activity against drug-resistant clinical UTI isolates. Screening of additional drug-resistant clinical isolates from UTI patients is underway.

